# The Reasons to Get Vaccinated: A Cross-Sectional Study on HPV Vaccination Adherence in a Northern Italian University

**DOI:** 10.3390/vaccines14010061

**Published:** 2026-01-04

**Authors:** Pier Mario Perrone, Riccardo Zanzi, Elia Biganzoli, Fabrizio Pregliasco, Silvana Castaldi

**Affiliations:** 1 Department of Clinical Sciences and Community Health, University of Milan, 20122 Milan, Italy; 2 Department of Clinical Sciences and Community Health, Laboratory of Medical Statistics, Biometry and Epidemiology “G. A. Maccacaro”, University of Milan, 20122 Milan, Italy; riccardo.zanzi@unimi.it; 3 Department of Biomedical and Clinical Sciences, Unit of Medical Statistics, Biometry and Epidemiology, University of Milan, 20157 Milan, Italy; elia.biganzoli@unimi.it; 4 IRCCS Ospedale Galeazzi-Sant’Ambrogio, 20157 Milan, Italy; fabrizio.pregliasco@unimi.it; 5 Department of Biomedical Sciences for Health, University of Milan, 20133 Milan, Italy; silvana.castaldi@unimi.it; 6 Quality Units, Fondazione Ca’ Granda Policlinico di Milano, 20122 Milan, Italy

**Keywords:** HPV vaccine, vaccination adherence, university population vaccine

## Abstract

Background/Objectives: Human papillomavirus (HPV) represents a major public health challenge due to its high prevalence and the complications arising from infection. The aim of the study was to investigate the reasons for adherence to the HPV vaccination campaign offered by the University of Milan to its students. Methods: A questionnaire, distributed via QR code, was utilized to investigate the motivations behind participation in the vaccination campaign, as well as the characteristics of the population participating in the vaccination campaign carried out at the University of Milan. Concurrently, a comprehensive analysis of the characteristics of students was also carried out at the vaccination sites where it was conducted, categorizing them into university hospitals and university campuses. Results: A comparison of vaccination sites revealed a significant disparity between hospitals and universities with regard to gender, age, and faculty. A higher average age (25 versus 24 years) and a higher prevalence of females (53.9% versus 51.1%) were observed in hospitals. The findings of the regression model demonstrate that demographic factors exert an influence on only two reasons for participation, with male gender proving a predictive factor for the response option entitled “It is a responsibility towards one’s partner(s)”. Furthermore, enrolment in a course of study has been found to correlate positively with the response option entitled “I have been convinced by advertising campaigns/friends/acquaintances”. Conclusions: A vaccination campaign implemented within educational institutions is a fundamental strategy for enhancing vaccination uptake rates among young population. Conversely, the utilization of health promotion interventions, such as pre-vaccination promotional campaigns, does not seem to be a pivotal factor in enhancing uptake.

## 1. Introduction

Human papillomavirus (HPV) infection is a major concern in public health, given its substantial clinical and economic implications. It is estimated that 831,204 new cases of cancer in women, resulting in 422,935 deaths worldwide in 2022, are attributable to HPV, despite the availability of a vaccine [1].

It is imperative to acknowledge the significant social, economic, and clinical ramifications that HPV infection can engender. Consequently, vaccination constitutes a pivotal instrument in the prevention of such consequences [2,3,4]. Currently, numerous types of vaccines directed against the main oncogenic strains are available, characterized by different administration schedules and coverage rates. Despite the extensive range of options available, however, coverage rates remain suboptimal for both underage and young adult populations [5,6].

A recent analysis of vaccination coverage rates in Italy reveals that only 62.2% of females and 49.9% of males born in 2006 have received the HPV vaccine [7], falling short of the 95% target rate set by the Ministry of Health [8]. In this regard, the analysis of the motivations behind both vaccine refusal and, even more so, the motivations behind adherence to vaccination campaigns becomes crucial. It is imperative to possess a comprehensive understanding of the factors that motivate adherence in specific populations, as emphasized in extant literature, in order to develop or export effective engagement models [6,7,9,10,11]. Whilst the majority of the aforementioned studies did not specifically address HPV vaccination, the implementation of tools designed to investigate the reasons behind adherence constitutes a fundamental intervention underlying vaccine promotion campaigns.

In this regard, the data presented in the extant literature appear to demonstrate a relatively consistent pattern regarding the factors and reasons underpinning vaccination adherence, irrespective of the specific type of vaccination for which the studies in question were conducted [2,12,13].

The objective of this study is to examine the factors that motivate individuals to participate in an HPV vaccination program among the student and staff population of a university in Northern Italy. Moreover, the study seeks to analyze the presence of distinct motivational patterns among different university demographics.

## 2. Materials and Methods

The University of Milan, in conjunction with the Welfare Department of the Lombardy Region and a tertiary hospital, has initiated a vaccination campaign targeted at students and university employees under the age of 27. The campaign was conducted in accordance with the most recent literature on the subject, which indicates that achieving optimal vaccination coverage necessitates the implementation of multiple strategies. Consequently, the working group determined that the implementation of two interventions would be necessary: a promotional and educational campaign, and the administration of the vaccination through an ad hoc and on-site outpatient center. The promotional and educational campaign, which had already been tested in previous influenza vaccination campaigns and which was being experimented with HPV vaccination for the first time, was launched in September 2024 and was carried out both through the University of Milan website, through a dedicated page, and through the University’s social channels. A dedicated online page was made accessible to all students and employees of the university. On this page, the commencement of the vaccination campaign was highlighted, along with detailed operational information on accessing the vaccination.

The promotional and educational campaign, conversely, was based on reels and posts dedicated to HPV in particular, describing its origin, the diseases it causes, the history of the vaccine, and prevention actions that can be implemented in the everyday context.

Ad hoc clinics were set up on the university campus from 18 to 22 November 2024. The hospitals participating in the campaign were the S. Paolo Hospital on 29 November, the Sacco Hospital on 5 and 6 December, the S. Carlo Hospital on 13 December and the scientific Institute for Research, Hospitalization and Health Care (IRCCS) San Donato Milanese, a research and university hospital directly involved in translational research and clinical activities both, on 20 December and 13 January. The hospital sites were identified to reach the student of the degrees in medical areas.

An anonymous, self-administered questionnaire was utilized to assess factors influencing vaccination adherence, in addition to age, gender, professional category, and area of activity. The aforementioned questionnaire, created through the EU Survey^®^ (version 1.5.3 released 12 March 2024) platform, was distributed via a link associated with the booking page and via a QR Code printed and distributed at the vaccination points for immediate completion during the waiting time after vaccination.

In consideration of the characteristics of the population under investigation and the specificity of the topic, namely the reasons behind the acceptance of the HPV vaccine rather than trust in or knowledge of this vaccine, the questionnaire used was not taken from scientific literature but was produced independently by the research group. The reasons underlying acceptance were developed on the basis of existing questionnaires not specifically related to HPV vaccination, which were supplemented and modified on the basis of the characteristics of the pathogen, including the fact that it is a sexually transmitted disease (STD) and therefore cannot be transmitted through the air.

The questionnaire used is included in the Supplementary Materials.

All the statistical analyses were conducted with the R software, release 4.4.2. A newly generated variable (age) was computed by considering the date of birth and the last day of the vaccination campaign. A summary statistic reporting the median and interquartile range was produced. Other variables were categorical, and for each class, the number of subjects and percentages are reported. Firstly, the data regarding gender, faculty, academic degree, and reasons for being vaccinated were compared using Fisher’s exact test or chi-squared test, as appropriate.

The Shapiro test was employed to evaluate the normality of the age variable, and the Kruskal–Wallis test was subsequently utilized for further analysis. Post hoc comparisons between the different paired vaccination sites were performed by means of Dunn’s test with Bonferroni’s method.

Multivariate logistic regression was then used to explore the relationships among the vaccination site, gender, faculty, academic degree, and reasons for being vaccinated. The results of the model were reported as odds ratios with a 95% confidence interval.

With regard to multivariate analysis, due to the significant imbalance in responses for several variables, including gender and course of study, the latter was not included among the independent variables analyzed, while the non-binary category was eliminated with regard to gender.

With regard to the rationales for vaccination, despite the marked imbalance in responses for this variable, with certain categories representing less than 5%, all categories were incorporated into the analysis to avoid the loss of potential motivations.

## 3. Results

Questionnaire responses were collected between November 2024 and March 2025. As illustrated in Table 1, the demographic data of respondents were categorized according to the vaccination site, with the distinction between hospital and university respondents. Notwithstanding the preponderance of female subjects in both cohorts, a slight predominance of females was observed in the hospital population (53.9%) in comparison to the university population (51.1%), exhibiting statistical significance. With regard to age, a statistically significant difference was observed. The median age was found to be higher in the hospital group than in the university group: 25 and 24 years, respectively. A statistically significant discrepancy was observed in the faculty distribution of the population. The medicine faculty was the most represented, with 42.4% of the population, followed by the science and technology faculty (12.2%) and other faculties (11.6%). The university population exhibited a slight range between 15 and 20% for medicine (18.5%); science and technology (16.8%); political, economic, and social sciences (15.7%); and humanities (17.6%).

A substantial discrepancy was identified in the composition of degree programs, with a preponderance of undergraduates (40.6%) and master’s degree students (26.2) within the university population. In contrast, the hospital population exhibited a higher proportion of single-cycle master’s students (32.2%). Finally, it is evident that the reasons for acceptance do not demonstrate a substantial statistical discrepancy between hospital and university students.

Table 2 presents the data on the reasons for vaccination. It is noteworthy that the predominant response among respondents to the questionnaire was that of considering vaccination as an effective preventive measure. This phenomenon is further substantiated by the response rates, which indicate that the predominant response of members of the various university courses is one of considering vaccination as an effective preventive measure. Furthermore, the second most common reason for choosing to be vaccinated in almost every faculty except Exercise and Sports Sciences is the fear of complications from a possible infection. In this faculty, the second most common reasons for vaccination are the risk of infection as a young person and responsibility towards one’s partner.

Supplementary Table S1 presents the factors that underpin the rationale for opting for vaccination. The regression model indicates that being male is a factor that favors the selection of the response option entitled “It is a responsibility towards one’s partner(s)” when asked to provide an explanation for their decision to adhere to vaccination recommendations (OR: 4.5, CI: 2.5–8.1). Conversely, the course of study does not appear to demonstrate a positive or negative correlation with the motivations for vaccine uptake, with the exception of “I have been convinced by advertising campaign/friends/acquaintances”, for which enrolment in a degree course correlates positively (OR: 71308, CI: 22637-224622; OR: 92562, CI: 16074-533009; OR: 163117, CI: 39788-668720) with participation in the campaign in comparison to employment as a postgraduate university employee.

## 4. Discussion

HPV is a significant public health concern due to its high prevalence and the oncogenic risk associated with infection [3,4]. In the context of infection, vaccination is considered a fundamental intervention strategy for reducing the overall burden of infection. Despite the proven effectiveness of vaccines and the importance of achieving adequate immunization rates, vaccine hesitancy remains a significant problem [6,11].

A multitude of studies have sought to ascertain the factors contributing to the suboptimal immunization rates observed among the younger demographic [14,15,16,17,18]. These studies have primarily focused on the evaluation of parental characteristics that may influence their propensity to prioritize the HPV vaccine [19,20,21]. Nevertheless, there is a paucity of studies that have sought to analyze both the characteristics associated with and the rationale for vaccine acceptance among young adults, who constitute one of the most significant demographics for HPV vaccination.

One of the earliest observed correlations with vaccination adherence is gender. In relation to this issue, the present study is in accordance with international research, which demonstrates a higher percentage of female subjects [22,23]. The preponderance of female cases has been documented in the extant literature for two principal reasons. Firstly, the perception of HPV as a female infection has been attributed to the historical association with cervical cancer. Secondly, the notion that male users of condoms could mitigate the risk of HPV transmission has been posited. This has resulted in a relative paucity of attention being paid to HPV vaccines among male populations.

In a similar manner, the present study is in accordance with international literature with regard to the impact of graduation programs on adherence to vaccination schedules. As has been demonstrated in a number of studies, there appears to be a correlation between enrollment in bachelor’s degree programs or single-cycle master’s programs and a heightened level of attention to vaccination [22,24]. In this regard, extant literature posits the relative novelty of the vaccine as the primary factor contributing to this discrepancy, a contention that finds substantiation in the existence of a more robust advisory campaign directed at the younger demographic.

Furthermore, the present study has sought to evaluate two particular components pertaining to the features associated with vaccination adherence. These components include the location of the vaccination and the relationship between the individual and the motivations for vaccination. In the context of the discussion around vaccination adherence and the role of the vaccination site in this regard, the extant literature has attempted to provide an assessment of the influence of the location of the vaccination on the adherence of the population to vaccination programs and the reasons for this. It should be noted, however, that the majority of studies in this field have focused on respiratory diseases for which vaccinations are available, such as the influenza pandemic and the SARS-CoV-2 virus [24,25,26,27]. The results of the study indicate three primary elements of discrepancy that are consistent with the findings reported in the extant literature.

The initial observation pertains to the demographic of the vaccinated population, which is presumably attributable to the presence of academic health professionals, albeit non-student members, such as medical residents or health researchers. In particular, it is reported in the literature that these categories, i.e., young healthcare professionals, pay more attention to vaccination [9,10,22]. The provision of the vaccine at the workplace may have resulted in the observed adherence, which is responsible for the observed age difference [9,28].

The second issue pertains to the nature of the course undertaken, with a significant proportion of undergraduates comprising the vaccinated population at the university’s headquarters. This phenomenon may be attributable to a number of factors. Firstly, the presence of a greater number of healthy professionals in single-cycle master’s programs or those who have already graduated and are working or studying at hospital sites may be a contributing factor to the observed decrease in the percentage of undergraduate students in this particular context. Secondly, it is possible that the heightened awareness of the HPV issue and the significance of vaccination as a preventative measure against infection, as highlighted in numerous studies, has led to a greater focus from the younger student population [15,22].

The third difference pertains to the faculty to which the vaccinated subjects belong. With regard to hospital venues, the figure confirms a high prevalence of students from the medical courses of study and professionals under the age of 26 employed by the university. This phenomenon can be elucidated by considering the context of the vaccine’s delivery, specifically the hospital delivery venue. This delivery method facilitates access for students and healthy professionals at their places of work. Conversely, it limits the accessibility of the vaccination to all professionals and students enrolled in other faculties. These individuals often benefit from the logistical advantages offered by central venues that are easily accessible from their usual places of study or work [9,29,30].

Finally, regarding the relationship between the field of study, which is healthcare rather than technology, and the reasons for participating in the vaccination campaign, a dichotomy of results can be observed, with a greater prevalence of students belonging to the faculty of medicine who adhered to the vaccination campaign on the basis of clinical motivations, including the perception of the vaccine as a preventive tool or the fear of complications due to HPV infection. These results align with the findings reported in the scientific literature, which indicate a higher level of knowledge and understanding of the technical aspects of vaccination and infection among health area students, particularly those in their final years of study, when compared to students or professionals from other academic backgrounds [22,31,32]. It is noteworthy that the literature frequently cites communication and advertising campaigns as a catalyst for enhanced adherence [33,34,35]. However, in this particular instance, these campaigns were cited by a minuscule number of subjects as a rationale for their participation in the vaccination campaign. This observation underscores the necessity of reevaluating the role of these interventions as non-essential components, particularly for age groups where peer communication may prove to be a more efficacious medium.

Despite its significance as the first study to examine the demographic characteristics and reasons behind adherence to an HPV vaccination campaign in an Italian university, the study is constrained by several limitations.

The main concern is the absence of a comparison group that would allow for an analysis of the characteristics and motivations behind the refusal of vaccination. This, combined with the relatively low rate of participation in the vaccination campaign, attributable to the shortage of available vaccines, represents a first obstacle to using these data to organize a new vaccination campaign in the same university setting. Secondly, the result is not generalizable to the entire national context. This phenomenon can be attributed to the characteristics of the university population on which the study was conducted. The population was characterized by a wide academic range, which consequently resulted in significant variability in adhesion and motivations behind adhesion between the various degree courses and faculties. Additionally, the presence of a substantial foreign student component, which is not commonly observed in all Italian universities, played a role in these findings. Finally, the territorial reach of the University of Milan, which facilitated access to workplaces for students and professionals regardless of their geographical location, represents an uncommon element in the Italian academic landscape. Consequently, the establishment of comparable and reproducible models within the same context is rendered challenging.

## 5. Conclusions

In conclusion, this study represents a fundamental step towards a greater understanding of the demographic characteristics and motivations behind adherence to HPV vaccination campaigns in young adults. Notwithstanding its limitations, the system facilitates the identification of population groups that can be targeted with specific, organized vaccination campaign models or specific communication campaigns.

It is precisely due to the centrality of the topics under discussion that this study is considered the initial step in a process that should result in the expansion of the working group to other university contexts or other working contexts with a high prevalence of young adults. The objective of this expansion is to enable the generalization of the results obtained at a national and international level.

## Figures and Tables

**Table 1:** Demographic data of HPV questionnaire responders.

	Hospitals N = 887	University N = 1694	*p*-Value
Age			
median (IQR)	25 (22–28)	24 (22–26)	<0.05
Gender (%)			
F	478 (53.9%)	865 (51.1%)	<0.05
M	408 (46.0%)	805 (47.5%)	
Not Binary	1 (0.1%)	24 (1.4%)	
Faculty (%)			
Other	103 (11.6%)	103 (6.2%)	<0.05
Law	36 (4.1%)	124 (7.3%)	
Medicine	376 (42.4%)	330 (19.5%)	
Veterinary Medicine	27 (3.0%)	26 (1.5%)	
Agricultural and Food Sciences	30 (3.3%)	82 (4.8%)	
Pharmacy	37 (4.2%)	104 (6.2%)	
School of Language Mediation and Intercultural Communication	21 (2.4%)	67 (3.9%)	
Science and Technology	108 (12.2%)	284 (16.8%)	
Exercise and Sports Sciences	5 (0.6%)	8 (0.5%)	
Political, Economic, and Social Sciences	77 (8.7%)	267 (15.7%)	
Humanities	67 (7.5%)	299 (17.6%)	
Programs (%)			
Other	198 (22.3%)	187 (11.0%)	<0.05
Masters’ Degree	124 (14.0%)	443 (26.2%)	
Single-cycle Master	286 (32.2%)	377 (22.2%)	
Undergraduate	279 (31.5%)	687 (40.6%)	
Acceptance Reasons (%)			
As a young person I am more at risk of acquiring an HPV infection	28 (3.2%)	79 (4.7%)	0.071
It is a responsibility towards my partner(s)	50 (5.6%)	79 (4.7%)	
I consider vaccination to be an effective prevention technique	708 (79.8%)	1307 (77.2%)	
I have been convinced by advertising campaigns/friends/acquaintances	3 (0.3%)	19 (1.1%)	
I fear the complications of HPV infection	86 (9.7%)	189 (11.1%)	
Other	12 (1.4%)	21 (1.2%)	

**Table 2:** Reasons to get vaccinated.

	As a Young Person I Am More at Risk of Acquiring an HPV Infection	It Is a Responsibility Towards My Partner(s)	I Consider Vaccination to Be an Effective Prevention Technique	I Have Been Convinced by Advertising Campaign/ Friends/ Acquaintances	I Fear the Complications of HPV Infection	Other
Faculty (%)						
Other	6 (2.9%)	5 (2.4%)	155 (75.2%)	1 (0.5%)	35 (17.0%)	4 (1.9%)
Law	12 (7.5%)	5 (3.1%)	124 (77.5%)	3 (1.9%)	14 (8.8%)	2 (1.3%)
Medicine	27 (3.8%)	27 (3.8%)	574 (81.3%)	3 (0.4%)	69 (9.8%)	6 (0.8%)
Veterinary Medicine	0	4 (7.5%)	42 (79.2%)	0	7 (13.2%	0
Agricultural and Food Sciences	2 (1.8%)	8 (7.1%)	88 (78.6%)	1 (0.9%)	10 (8.9%)	3 (2.7%)
Pharmacy	3 (2.1%)	1 (0.7%)	119 (84.4%)	0	15 (10.6%)	3 (2.1%)
School of Language Mediation and Intercultural Communication	6 (6.8%)	4 (4.5%)	69 (78.4%)	0	8 (9.1%)	1 (1.1%)
Science and Technology	16 (4.1%)	27 (6.9%)	308 (78.6%)	6 (1.5%)	31 (7.9%)	4 (1.0%)
Exercise and Sports Sciences	1 (7.7%)	1 (7.7%)	11 (84.6%)	0	0	0
Political, Economic and Social Sciences	15 (4.4%)	27 (7.8%)	250 (72.7%)	4 (1.2%)	41 (11.9%)	7 (2.0%)
Humanities	19 (5.2%)	20 (5.5%)	275 (75.1%)	4 (1.1%)	45 (12.3%)	3 (0.8%)

## Data Availability

The raw data supporting the conclusions of this article will be made available by the authors upon request.

## Supplementary Materials

The following supporting information can be downloaded at: https://www.mdpi.com/article/10.3390/vaccines14010061/s1, Table S1: Factors influencing reasons for vaccination uptake
