# Supplementary material for: The Reasons to Get Vaccinated: A Cross-Sectional Study on HPV Vaccination Adherence in a Northern Italian University
Source: Vaccines (Basel). 2026 Jan 4;14(1):61. doi: 10.3390/vaccines14010061 (PMC12846412; doi:10.3390/vaccines14010061)
Supplement: Supplementary file 1 [file vaccines-14-00061-s001.zip › vaccines-4063854-supplementary.pdf]

Supplementary Table S1 Factors influencing reasons for vaccination uptake

Characteristic	OR	95% CI	p-value
**It is a responsibility towards my partner(s)**			
Sex			
M	4.50	2.50, 8.10	<0.001
F			
Age	1.02	0.95, 1.09	0.6
Programs			
Other			
Undergraduated	1.64	0.60, 4.49	0.3
Masters' Degree	2.48	0.89, 6.94	0.083
Single-cycle Master	1.85	0.62, 5.55	0.3
Vaccination Site			
Hospital			
University	0.49	0.28, 0.88	0.016
**I consider vaccination to be an effective prevention technique**			
Sex			
M	0.98	0.66, 1.45	>0.9
F			
Age	1.03	0.97, 1.08	0.3
Programs			
Other			
Undergraduated	0.91	0.45, 1.87	0.8
Masters' Degree	1.36	0.65, 2.83	0.4
Single-cycle Master	2.17	0.98, 4.80	0.055
Vaccination Site			
Hospital			
University	0.69	0.44, 1.09	0.11
**I have been convinced by advertising campaign/friends/acquaintances**			
Sex			
M	1.25	0.48, 3.24	0.7
F			
Age	0.92	0.76, 1.12	0.4
Programs			
Other			
Undergraduated	71,308	22,637, 224,622	<0.001
Masters' Degree	92,562	16,074, 533,009	<0.001
Single-cycle Master	163,117	39,788, 668,720	<0.001
Vaccination Site			
Hospital			
University	1.97	0.53, 7.31	0.3
**I fear the complications of HPV infection**			
Sex			
M	0.69	0.44, 1.09	0.12
F			
Age	1.03	0.97, 1.09	0.4
Programs			
Other			
Undergraduated	0.49	0.22, 1.09	0.080
Masters' Degree	1.22	0.54, 2.75	0.6

Single-cycle Master	1.28	0.53, 3.08	0.6
Vaccination Site			
Hospital			
University	0.84	0.50, 1.41	0.5
**Other**			
Sex			
M	1.14	0.51, 2.55	0.8
F			
Age	1.07	1.00, 1.15	0.056
Programs			
Other			
Undergraduated	0.62	0.15, 2.48	0.5
Masters' Degree	2.29	0.63, 8.30	0.2
Single-cycle Master	2.16	0.53, 8.74	0.3
Vaccination Site			
Hospital			
University	0.63	0.27, 1.50	0.3
